# *Crataegus monogyna* Jacq., *Sorbus aria* (L.) Crantz and *Prunus spinosa* L.: From Edible Fruits to Functional Ingredients: A Review

**DOI:** 10.3390/foods14132299

**Published:** 2025-06-28

**Authors:** Cristina Tamayo-Vives, María Úbeda, Patricia Morales, Patricia García-Herrera, María Cortes Sánchez-Mata

**Affiliations:** 1 Department of Nutrition and Food Science, Faculty of Pharmacy, Complutense University of Madrid, Plaza Ramón y Cajal s/n, 28040 Madrid, Spain; critamay@ucm.es (C.T.-V.); patricia.morales@farm.ucm.es (P.M.); cortesm@ucm.es (M.C.S.-M.); 2 Cunimad-University Center Attached to the University of Alcalá (UAH), Calle de García Martín 21, 28224 Pozuelo de Alarcón, Spain; ubedacantera@gmail.com

**Keywords:** biological activities, bioactive compounds, technological uses, Mediterranean Diet, hawthorn, whitebeam, blackthorn

## Abstract

Plants have historically served as key sources of nutrition and popular medicine, which persists in current applications. The increasing demand for natural bioactive compounds has intensified the incorporation of plant-derived ingredients in both the food and pharmaceutical industries. This narrative review focuses on the fruits of *Crataegus monogyna* Jacq., *Sorbus aria* (L.) Crantz, and *Prunus spinosa* L. (Rosaceae), traditionally utilized in Europe and characterized by a high content of phenolic compounds, flavonoids, and anthocyanins. These metabolites are associated with antioxidant, anti-inflammatory, and cardioprotective properties. The available literature on their phytochemical profiles, biological activities, and integration into the Mediterranean Diet is critically assessed. Evidence supports their potential as functional food components. Despite encouraging in vitro results, the scarcity of in vivo and clinical studies limits the translational potential of these findings. Further research is warranted to validate their efficacy and safety in human health. This review underscores the value of integrating traditional ethnobotanical and ethnopharmacological knowledge with contemporary scientific research to explore novel applications of these underutilized wild fruits.

## 1. Introduction

Among the *Rosaceae* family, *Crataegus monogyna* Jacq., *Sorbus aria* (L.) Crantz and *Prunus spinosa* L. have been traditionally consumed in rural Mediterranean regions, during the autumn season, when they are fully ripe (September–December) [1,2,3]. *C. monogyna* is known for its cardioprotective, digestive, and sedative properties, attributed to its high content of flavonoids, phenolic acids and essential oils [1,4,5,6,7,8]. *S. aria* is used as a food ingredient and traditional remedy due to its diuretic, anti-inflammatory, and vasodilatory properties, with its phenolic-rich fruits often processed into jams, syrups and alcoholic beverages [9]. *P. spinosa* is valued for its diuretic, antimicrobial and antioxidant activities. It has been widely studied for its potential in managing diabetes, cardiovascular diseases and neurodegenerative conditions, with its fruits traditionally consumed in various forms, such as juices, wines and tinctures [10,11,12] (Table 1).

In recent decades, natural antioxidants such as carotenoids and anthocyanins have emerged as preferred alternatives to synthetic additives, driven by increasing consumer demand for natural, organic, and plant-based products with health-promoting properties [1,13,14]. This trend has contributed to the growing development of functional foods and plant-based dietary supplements that blur the line between nutrition and medicine, supporting self-care and preventive health strategies [15,16]. Within this context, the integration of *Crataegus monogyna*, *Sorbus aria*, and *Prunus spinosa* into modern diets represents a convergence of traditional ethnobotanical knowledge and contemporary nutritional science. However, further research is needed to validate their functional properties and optimize their application in both the food industries [17,18].

The general objective of this narrative review is to provide an overview of the scientific evidence on the use and effects of *C. monogyna*, *S. aria*, and *P. spinosa* fruits as part of the diet or as ingredients in foods or dietary supplements within the Mediterranean Diet. To achieve this objective, the following specific objectives are intended: (i) to detail the chemical composition in terms of phenolic compounds and related components present in these edible wild fruits; (ii) to explore, through in vitro and in vivo studies, the health benefits offered by these fruits due to the aforementioned bioactive compounds; and (iii) investigate the feasibility of incorporating these fruits into the current diet, whether as foods, food ingredients, or dietary supplements.

## 2. *Crataegus monogyna* Jacq. Fruits

*Crataegus monogyna* Jacq., belonging to the subfamily Amygdaloideae within the *Rosaceae* family, is distributed worldwide, although it is considered native to Europe, particularly in areas bordering the Mediterranean basin, and can also be found on the northern coasts of Africa. It is commonly known as hawthorn and is a semi-evergreen shrub or small tree ranging from 5 to 10 m in height, usually equipped with thorns. Its fruits are commonly known as hawthorn berries and are defined as subglobose or cylindrical pomes (5.5–10 × 4.4–9.5 mm) with one to three seeds inside, turning red when ripening in autumn, and with a greenish calyx [13,14,19].

### 2.1. Chemical Composition of C. monogyna Fruits

*C. monogyna* is rich in phenolic compounds, including phenolic acids, flavonoids, proanthocyanins, anthocyanins, and catechins, along with tocopherols, ascorbic acid, β-carotene, saturated and polyunsaturated fatty acids, and essential inorganic elements such as Cu, Fe, Mg, Mn and Zn. The bioactive properties of these fruits are primarily attributed to their phenolic and organic acid content (Table 2) [20,21]. Compounds were identified by chromatographic methods (LC-DAD, GC-MS, etc.)

Organic acid composition varies among studies; Morales et al. (2013) [21] identified malic, citric and oxalic acids in high concentrations, whereas other researchers reported differing acid predominance, including succinic, tartaric and fumaric acids [22,23,24].

Regarding phenolic acids, *C. monogyna* contains hydroxycinnamic acids (chlorogenic, neochlorogenic, ferulic, caffeic, sinapic, p-coumaric and q-coumaric acids) and hydroxybenzoic acids (quinic, protocatechin, salicylic, syringic, gallic, vanillic, ellagic and their derivatives). These compounds contribute significantly to its antioxidant potential [23,24,25,26,27,28,29,30,31,32,33]. However, some studies did not detect certain acids such as caffeic, syringic, p-coumaric, ferulic and sinapic [34].

**Table 2:** Organic acids and phenolic compounds found in the fruits of *Crataegus monogyna* Jacq.

Compound Families		Compounds	Reference
Organic acids		Malic	
		Citric	
		Succinic	
		Ascorbic	[21,22,23,24]
		Dehydroascorbic	
		Oxalic	
		Fumaric	
		Tartaric	
Phenolic acids	Hydroxycinnamic acids	Chlorogenic acid	
		Neochlorogenic acid	
		Ferulic acid	
		Caffeic acid	[23,24,25,26,27,28,29,30,31,32,33]
		Sinapic acid	
		p-Coumaric acid	
		q-Coumaric acid	
	Hydroxybenzoic acids	Quinic acid	
		Protocatechinic acid	
		Salicylic acid	
		Syringic acid	
		Gallic acid	[23,24,25,26,27,28,29,30,31,32,33]
		Vanillic acid	
		3-hydroxybenzoix acid	
		4-hydroxybenzoix acid	
		3,4-hydroxybenzoix acid	
Flavonoids	Flavan-3-ols	(±)-catechins	[25,32,33,35]
		(±)-epicatechin	
	Flavanols	Procyanidin B2	
		Procyanidin B4	
		Procyanidin B5	[25,26,27,28,29,30,32,33,34,35]
		Procyanidin C1	
		Procyanidin D1	
		Oligomeric proanthocyanidins	
	Flavonols	Hyperoside	
		Vitexin	
		Vitexin-2″-O-rhamnoside	
		Acetyl vitexin-2″-O-rhamnoside	
		Rutin	
		Quercetin	
		Quercetin-3-O-glucoside (Isoquercetin)	[25,26,27,28,29,30,32,33,34,35]
		Quercetin-3-O-rhamnoside	
		Quercetin-3-O-rutoside	
		Kaempferol-3-O-glucoside	
		Myricetin	
	Anthocyanidins	Cyanidin-3-O-glucoside	
		Cyanidin-3-O-arabinoside	
		Cyanidin-3-galactoside	
		Malvidin 3-O-(4‴-coumaroyl) rutinoside 5-O-glucoside	
		Peonidin 3-O-(4‴-coumaroyl) rutinoside 5-O-glucoside	[36,37,38]
		Petunidin 3-O-(4‴coumaroyl) rutinoside 5-O-glucoside	
		Malvidin 3-O-(4‴-coumaroyl) rutinose	

Flavonoid analysis using RP-HPLC revealed the presence of flavan-3-ols ((±)-catechins and (±)-epicatechin), procyanidins B2, B4, B5, C1 and D1, along with oligomeric proanthocyanidins. These compounds undergo oxidation, forming dimers and oligomers. The main flavonoids identified include hyperoside, vitexin, rutin, quercetin, isoquercetin, myricetin and their glycosylated derivatives [25,26,27,28,29,30,32,33,34,35]. Stoenescu et al. (2022) [34] did not detect quercetin in their analysis.

The red pigmentation of *C. monogyna* fruits is attributed to anthocyanins, with cyanidin-3-O-glucoside and cyanidin-3-O-arabinoside being predominant [36]. Other studies identified additional anthocyanins such as cyanidin-3-galactoside, malvidin, peonidin, and petunidin derivatives [37]. Similarly, Tamayo-Vives et al. (2023) [38] tentatively identified cyanidin-O-hexoxide, cyanidin-3-O-glucoside, peonidin-O-hexoxide and other cyanidin derivatives in *C. monogyna* fruits.

C. monogyna fruits show TPC values ranging from 1500 to 2800 mg gallic acid equivalents (GAE)/100 g dry weight (dw), depending on the extraction method and ripening stage [30,38]

### 2.2. Biological Activities of C. monogyna Fruits: In Vitro and In Vivo Studies

Numerous scientific articles have focused on the study of bioactivities of *C. monogyna* fruits, in which its antioxidant and other bioactivities have been tested (Table 3).

#### 2.2.1. In Vitro Studies

- **Antioxidant effect**

The antioxidant activity of *C. monogyna* extracts has been extensively studied using various in vitro methodologies, including DPPH radical scavenging, FRAP, β-carotene bleaching inhibition, lipid peroxidation inhibition, TEAC, TBARS, and OxHLIA assays. Among these, the DPPH assay is commonly used to assess the free radical scavenging capacity of plant extracts [39,40]. The lyophilized extract of *C. monogyna* has demonstrated significant antioxidant potential, with ethanol and methanol extracts showing scavenging activities of 74.90% and 66.86%, respectively [7,29,41,42]. Additionally, the TEAC assay has been widely applied to evaluate total radical elimination capacity, particularly against the ABTS•+ radical cation [7,29].

Studies have also highlighted *C. monogyna*’s ability to inhibit lipid peroxidation, demonstrated through β-carotene, linoleic acid and TBARS assays [21]. Segmenoglu et al., 2024 [42] evaluated the antioxidant capacity of both pulp and seed using multiple assays, including ABTS•+, DPPH, CUPRAC, β-carotene, linoleic acid, and metal chelation. Results indicate a strong correlation between the antioxidant potential and the phenolic and flavonoid content of ripe fruit extracts [29,39,40].

Beyond antioxidant activity, *C. monogyna* has also demonstrated genotoxic and antigenotoxic effects. Barreira et al. (2015) [43] assessed ethanolic extracts in lymphocytes from healthy donors using the comet assay, revealing significant differences across plant parts and concentrations. Furthermore, Radi et al. (2023) [44] evaluated the antioxidant activity of aqueous extracts and their acute toxicity, finding strong antioxidant capacity (IC50 9.23 mg/mL by DPPH, 8.32 mg/mL by FRAP) with no toxic effects in albino mice.

Moreover, *C. monogyna* extracts exhibited remarkable free radical scavenging activity (>70% by DPPH), highlighting their potential in mitigating oxidative stress-related damage. This activity is largely attributed to phenolic compounds such as catechins and flavonoids, which contribute significantly to their overall antioxidant properties [45].

- **UV photoprotective effect**

The antioxidant compounds contained in *C. monogyna* presented a high potential for protection against both UVB and UVA radiation, which were experimentally tested in vitro; as a result, the incorporation of polyphenolic compounds, such as antioxidants, photostabilizers, and UV filters, into sunscreen formulations represents a promising strategy for the development of broad-spectrum sunscreen products with enhanced efficacy and safety [46]. The possibility of developing such an effect through oral ingestion, as a contribution to oxidative balance in different parts of the body, including the skin, could be further investigated.

- **Antitumor effect**

The antitumor capacity of *C. monogyna* extracts is strongly related to the antiproliferative and apoptotic effects of phenolic compounds. This was tested in four human tumour lines: NCI-H460, HepG2, HeLa, and MCF-7. HPLC-DAD-ESI/MS analysis of the extracts revealed the predominance of flavonoids. An interesting fact is that, in addition to not producing toxicity in non-tumour cells, the aqueous extract of *C. monogyna* is able to exert a protective effect against the toxicity caused by doxorubicin, a broad-spectrum chemotherapeutic drug whose use has been restricted [47].

Mraihi et al. (2015) [36] focused on characterizing the phytochemical composition of wild Tunisian edible hawthorn fruits, which are rich in flavonoids but have limited characterization. Using liquid chromatography coupled with mass spectrometry (LC-ESI/MS), researchers identified nine flavonoid glucosides, including isoquercitin, quercitin-digalactoside, and kaempherol-3-glucoside, among others. The MTT assay showed that *C. monogyna* was highly effective against the MCF-7 tumour cell line. The results indicated that hawthorn fruits contain significant amounts of diverse flavonoids, suggesting potential health benefits and applications in the pharmaceutical industry owing to their ability to inhibit tumour growth.

The *C. monogyna* extract significantly inhibited the proliferation of A549 lung cancer cells in a concentration-dependent manner, with greater efficacy observed at 200 μg/mL. Compounds such as catechin and quercetin likely contribute to their antitumor properties, highlighting their potential as natural anticancer agents [42].

- **Anticoagulant effect**

To deepen the content of the extracts, the polyphenol-polysaccharide conjugates were isolated and characterized using several spectrophotometric methods such as GLC-MS, FT-IR and NMR techniques. It was observed that the content was polyphenol matrices, including some flavonoids and poorly esterified polysaccharides rich in galacturonic acid, capable of increasing the blood coagulation time at very low concentrations (31.25 μg/mL), as verified in vitro, although the flower extract showed greater selectivity. Both purified products have proven to be the effective inhibitors of the process of clot formation by the intrinsic pathway of the coagulation cascade, with activity of 2.49 IU/mg for flower and of 1.23 IU/mg for fruits, when compared to the activity of 6th ISUH (200.5 IU/mg), the test for 6th International Standard for Unfractionated Heparin (WHO International Standard, NIBSC code: 07/328) [48].

- **Antimicrobial effect**

The antibacterial activity of *C. monogyna* fruit extracts is moderate, especially against gram-positive bacteria such as *Listeria monocytogenes*, *Micrococcus flavus* and *Bacillus subtilis* [49]. This agrees with Özderin et al. (2024) [50], who found a considerable inhibitory effect on *B. subtilis* and *Staphylococcus aureus*, with minimal effects against *Escherichia coli* and *Pseudomonas aeruginosa*. Furthermore, they reported that the activity against the fungus *Candida albicans* was more relevant than the antibacterial activity, especially in dried hexane extracts.

Ethanol extracts of *C. monogyna* fruits exhibit a stronger bactericidal effect than streptomycin, especially against *M. flavus*, *B. subtilis*, and *L. monocytogenes*, with inhibition rates of 80.0%, 62.2%, and 60.7%, respectively [7].

In addition, hawthorn extracts, which contain hyperoxide and procyanidins as the main compounds, showed antibacterial activity, particularly against gram-positive bacteria such as *M. flavus*, *B. subtilis*, and *L. monocytogenes*, with no effect against *C. albicans* [35].

The *C. monogyna* extract showed moderate inhibition zones against bacteria such as *S. aureus*, *Klebsiella pneumoniae*, and *Campylobacter jejuni*. However, these levels are insufficient to consider the extract an effective antimicrobial agent against resistant pathogens by clinical standards [42].

- **Hypoglycemic effect**

The presence of α-glucosidase in the small intestine is particularly important for the digestion of dietary carbohydrates. Inhibitors of this enzyme slow the digestion of carbohydrates, thereby slowing glucose absorption and postprandial hyperglycaemia. The fruit extract of *C. monogyna* exhibited exceptional α-glucosidase inhibitory activity (IC50 = 0.56 mg/mL), which has been attributed mainly to hydroxycinnamic acids [29].

In another study, *C. monogyna* extracts obtained by accelerated solvent extraction and laser irradiation extraction yielded good yields. The extract obtained by accelerated solvent extraction inhibited α-amylase with an IC50 of 0.44 μg/mL and α-glucosidase with an IC50 of 77.1 μg/mL. Additionally, the extract stimulated insulin secretion in β-TC-6 cells, suggesting that *C. monogyna* fruits are a source of bioactive compounds with antioxidant and anti-diabetic properties [51].

Also, in the Radi et al. (2023) [44] study, *C. monogyna* aqueous extracts demonstrated significant antihyperglycemic effects, inhibiting pancreatic α-amylase with an IC50 of 0.070 mg/mL. Catechins and rutin are potent inhibitors of α-amylase. These results suggest that *C. monogyna* can be used as a dietary supplement for diabetes prevention and treatment.

- **Cardioprotective effect**

The ethanol extract of *C. monogyna* produced dose-dependent inhibition of cathepsin S, with a maximum inhibition of enzyme activity of 71.7%. Although cathepsin inhibitors, including cathepsin S inhibitors, have been extensively studied for their anti-inflammatory and antitumor properties, very few researchers have investigated the medicinal plants used in the treatment of cardiovascular disease for cathepsin S inhibition [52].

The ethanolic extract of *C. monogyna* may provide a protective cardiovascular effect, either by preventing atherosclerotic activity or by attenuating the progression of existing lesions through reporting mechanisms with cathepsin S inhibitors [52].

These effects were attributed to the presence of phenolic compounds extracted using polar solvents. Ethanol is an excellent solvent for polyphenol extraction, although both ethanol and distilled water are polar. Therefore, it is possible that the inhibitory effects of cathepsin S from *C. monogyna* may be due to the presence of phenolic compounds [52].

In addition, hawthorn preparations, such as propranolol, were found to display negative chronotropic effects in cultured neonatal murine cardiomyocytes, but without causing arrhythmias. These effects were not due to beta-adrenergic receptor blockade, suggesting a different mechanism. Recent studies have shown that hawthorn extract decreases the contraction rate of cardiomyocytes via muscarinic receptor activation [35].

Flavonoids in hawthorn (luteolin-7-glucoside, hyperoside, and rutin) were tested in the guinea pig heart. At high concentrations (0.5 mmol/L), they increased coronary flow (by about 186%, 66%, and 66%, respectively) and relaxation velocity (by about 104%, 62%, and 73%, respectively), suggesting potential benefits on cardiac function [35].

- **Immunomodulatory effect**

Polysaccharides from hawthorn fruit (*Crataegus* spp.), particularly the AF2 fraction, are rich in galacturonic acid, giving them pectic-like structural features and high uronic acid content. AF2 demonstrates significant immunostimulatory effects by enhancing macrophage activity, increasing pro-inflammatory cytokines (IL-1β, IL-6, and TNF-α), and activating the NF-κB signalling pathway via toll-like receptor-4 (TLR4). Thus, AF2 is the key component of hawthorn fruit polysaccharides (HFP) responsible for their immunomodulatory properties. These findings highlight HFP, particularly AF2, as a promising natural source of functional foods with immune-enhancing benefits [53].

- **Anti-inflammatory effect**

The triterpene fraction from hawthorn was tested for its anti-inflammatory activity in vitro by inhibiting phospholipase A2. The results showed a weak inhibitory capacity, indicating limited in vitro anti-inflammatory effects [35].

The *C. monogyna* extract exhibited notable anti-inflammatory effects by reducing the production of inflammatory cytokines, such as TNF-α and IL-6. These results highlight its potential for mitigating inflammation-related conditions, with phenolic compounds playing a crucial role in suppressing excessive inflammatory responses [45].

- **Neuroprotective effect**

Hawthorn flavonoids decreased reactive oxygen species production in brain homogenates in a dose-dependent manner. They also reduce the levels of inflammatory cytokines and demonstrate protective effects in an ischemia/reperfusion injury model. This suggests that hawthorn can protect the brain against ischemia-reperfusion and improve behaviour through mechanisms involving a reduction in lipid peroxidation and nitric oxide levels by decreasing peroxynitrite formation. In addition, hawthorn pulp extracts caused central nervous system depression, leading to decreased exploratory behaviour and locomotor activity in experimental animals. This indicates the potential sedative effects of hawthorns [35].

**Table 3:** In vitro and in vivo studies on bioactive compounds in *C. monogyna* fruits.

Effects	Study	Solvent	Compounds Responsible for Activity	References
**In vitro studies**				
Antioxidant	DPPH radical scavenging effect, ferric reducing antioxidant power (FRAP), inhibition of β-carotene bleaching and inhibition of lipid peroxidation of brain cells.	Methanol	Phenolic compounds, flavonoids	[39]
	Limiting the formation of free radicals.	Supercritical CO_2_	Not detected	[40]
	DPPH radical scavenging effect, Trolox equivalent antioxidant capacity (TEAC), ferric reducing antioxidant power (FRAP), thiobarbituric acid reactive substances assay (TBARS), inhibition of oxidative haemolysis (OxHLIA).	Ethanol 70%	Phenolic compounds, flavonoids	[29]
	DPPH radical scavenging effect.	Ethanol	Phenolic compounds	[41]
	Antioxidant activities by: ABTS•+ and DPPH FRAP Brain cells homogenates.	Ethanolic extract Methanolic extract Ethanolic extract	Phenolic compounds	[7]
	Free radical scavenging activities of 74.90% for the ethanol extract and 66.86% for the methanol extract.	Ethanol Methanol	Phenolic compounds	[42]
	Lipid peroxidation inhibition was measured by in vitro methods such as β-carotene linoleic acid assay and TBARS assays.	Ethanol	Phenolic compounds	[21]
	ABTS•+ radical cation, DPPH radical scavenging assay, CUPRAC, β-carotene linoleic acid assay, and metal chelation using EDTA as the reference compound.	Ethanol	Flavonoids and procyanidin	[50]
	Genotoxicity and antigenotoxicity in lymphocytes using *C. monogyna* extracts, evaluated through the comet assay, considering metabolic activation with S9 and the bioactivity potential in natural matrices.	Ethanolic extracts	Polyphenols, flavonoids, and other antioxidants	[43]
	The extract exhibited strong antioxidant activity (IC50 9.23 mg/mL by DPPH, 8.32 mg/mL by FRAP) and showed no toxicity in albino mice.	Aqueous extracts	Phenolic compounds	[44]
	Using DPPH assays, it showed a free radical scavenging rate above 70%, indicating strong potential to neutralize reactive oxygen species (ROS).	Aqueous extracts	Phenolic compounds, such as catechins and flavonoids	[45]
UV photoprotection	Photoprotector at the cellular and mitochondrial level.	Chloroform/Alkaline Extraction	Phenolic compounds	[46]
Antitumor	Inhibition of HepG2 (hepatocellular carcinoma), NCI-H460 (non-small cell lung cancer), HeLa (cervical carcinoma), and MCF-7 (breast adenocarcinoma).	Methanol 80%	Phenolic compounds, flavonoids	[37]
	Strong effectiveness against MCF-7 tumour cells.	Ethanol/acidified water HCl 1.5 N	Flavonoid glucosides	[36]
	Inhibition of the proliferation of A549 lung cancer cells in a concentration-dependent manner, with greater efficacy observed at 200 micrograms per millilitre.	Ethanolic extracts	Catechin and quercetin	[42]
Anticoagulant	Prolong the activated partial thromboplastin time test and the prothrombin time test.	Alkaline extraction	Polyphenols, polysaccharides	[48]
Antibacterial	Inhibition of *Listeria monocytogenes*, *Micrococcus flavus* and *Bacillus subtilis*	Ethanol 70%	Phenolic compounds, flavonoids	[49]
	Inhibition of *Candida* albicans, *Staphylococcus aureus* and *Bacillus subtilis*.	Ethanol 70%	Phenolic compounds, flavonoids	[50]
	Bactericidal effect compared with streptomycin, especially against *Micrococcus flavus*, *Bacillus subtilis*, *and Listeria monocytogenes*.	Ethanol	Flavonoids	[7]
	Antibacterial activity, particularly against Gram-positive bacteria like *Micrococcus flavus*, *Bacillus subtilis*, and *Listeria monocytogenes*, with no effect against *Candida albicans*.	Ethanolic extract	Hyperoxide and procyanidins	[35]
	*C. monogyna* extract showed moderate inhibition zones against bacteria such as *Staphylococcus aureus*, *Klebsiella pneumoniae*, and *Campylobacter jejuni*.	Ethanol Methanol	Phenolic compounds	[42]
Hypoglycemic	Inhibition of fungal α-glucosidase.	Ethanol 70%	Phenolic acids (hydroxycinnamic acids), flavonoids	[29]
	Inhibition of α-amylase with an IC50 of 0.44 micrograms per millilitre and α-glucosidase with an IC50 of 77.1 micrograms per millilitre. Additionally, the extract stimulated insulin secretion in β-TC-6 cells.	Accelerated solvent extraction and laser irradiation extraction	Phenolic compounds	[51]
	Antihyperglycemic effects, inhibiting pancreatic α-amylase with an IC50 of 0.070 mg/mL.	Aqueous extracts	Catechin and rutin	[44]
Cardioprotective	Dose-dependent inhibition (200 μg/mL) of Cathepsin S, with a maximum inhibition of enzyme activity of 71.7%.	Ethanolic extract	Phenolic compounds	[52]
	Negative chronotropic effects in cultured neonatal murine cardiomyocytes via muscarinic receptor activation. Also, increase coronary flow by about 186%, 66%, and 66%, respectively, and relaxation velocity by about 104%, 62%, and 73%, respectively.	Ethanolic extract	Flavonoids	[35]
Immunomodulation	Galacturonic acid-rich AF2 fraction enhances immune responses by activating macrophages and the NF-κB pathway via TLR4.	Not determined	Uronic acid	[53]
	Anti-inflammatory activity in vitro by inhibiting phospholipase A2.	Ethanolic extract	Flavonoids	[35]
	Reduction of the production of inflammatory cytokines like TNF-α and IL-6.	Aqueous extracts	Phenolic compounds	[45]
Neuroprotective	Neuroprotective effects by reducing ROS, inflammation, lipid peroxidation, and peroxynitrite formation, alongside sedative properties decreasing locomotor activity. Additionally, *C. monogyna* extracts show anti-platelet effects by inhibiting ADP-induced platelet aggregation and serotonin release.	Ethanolic extract	Flavonoids	[35]
Anti-obesity	Considerable potential in reducing lipid accumulation in adipose cells, with a reduction of approximately 40% in lipid content.	Aqueous extracts	Phenolic compounds	[45]
**In vivo studies**				
Anti-inflammatory	72.4% decrease in inflammation in rats.	70% ethanol	Terpenoids	[49]
	Reduction of hind-paw edema by 61.5% and 52.5% at 3 and 5 h at the highest dose of 40 mg/kg, and peritoneal leucocyte infiltration by 41.9%, 64.7%, and 89.4% at doses of 10, 20, and 40 mg/kg, respectively. Also, it has an effect in the carrageenan-induced rat paw edema model, showing 72.4% effectiveness at a dose of 200 mg/kg.	Ethanolic extract	Terpenoids	[35]
Antithrombotic	Inhibition of platelet aggregation in mice.	50% ethanol	Phenolic compounds and flavonoids	[54]
	Reduction of the length of tail thrombosis by 61.5% and 52.5% at 3 and 5 h, at the highest dose of 300 mg/kg.	Ethanolic extract	Phenolic compounds and flavonoids	[35]
Cardioprotective and antiarrhythmic	Protective effect of myocardial dysfunction and occurrence of myocardial infarction in rats.	Supercritical CO_2_	Phenolic compounds and flavonoids	[40]
	Vasodilator effect and inotropic effect in guinea pigs.	Alkaline extraction	Phenolic compounds and flavonoids	[48]
	Antiarrhythmic effect in Wistar rats after the administration of 4 mg/kg for 60 min by intravenous injection.	Ethanol	Flavonoids	[7]
Analgesic	Depression of the central nervous system. Analgesic effects mediated by the endogenous opioid system.	80% ethanol	Flavonoids, procyanidins, organic acids, tannins and triterpene derivatives	[35]
Gastroprotective	Significant dose-dependent gastroprotective activity comparable to that of the reference drug ranitidine.	70% ethanol	Flavonoids	[49]
	In a rat model of ethanol-induced acute stress ulcer, hawthorn extract demonstrated gastro-protective activity comparable to that of ranitidine.	80% ethanol	Flavonoids, procyanidins, organic acids, tannins and triterpene derivatives	[35]
Immunomodulatory effect	Increase in humoral immune response and lymphocyte subsets.	Ethyl acetate	Phenolics	[7]
Neuroprotective effect	Anti-ACE activity (IC value of 335.00 μg/mL). Also, analgesic effects that were antagonized by naloxone, suggesting opioid receptor-mediated analgesic effects.	Hydroethanolic extract	Oleanolic acid	[35]

The *C. monogyna* extract was investigated for its potential as a therapeutic agent for migraine. In vitro conditions inhibited ADP-induced human platelet [14C]5-hydroxytryptamine release and significantly inhibited ADP-induced platelet aggregation. The authors concluded that further studies are needed to elucidate the compounds responsible for these antiplatelet effects and to determine their exact mechanism of action [35].

- **Anti-obesity effect**

Hawthorn extract showed considerable potential in reducing lipid accumulation in adipose cells, with a reduction of approximately 40% in the lipid content. This suggests that it can regulate lipid metabolism, making it a promising candidate for addressing obesity-related issues [45].

#### 2.2.2. In Vivo Studies

- **Anti-inflammatory effect**

Oral administration of doses, 50, 100, and 200 mg/kg of *C. monogyna* extracts to rats with edema led to 20.8, 23.0, and 36.3% reduction in edema, respectively. That demonstrated the anti-inflammatory effect of this plant, compared to the effect produced by indomethacin, a drug used as a positive control. The extract produced a 72.4% decrease in inflammation compared with 50% achieved by the control. This activity is associated with the presence of triterpenes, particularly cycloartenol [49].

In rats, a triterpene fraction from hawthorn reduced hind-paw edema by 61.5% and 52.5% at 3 and 5 h (at the highest dose of 40 mg/kg), and peritoneal leukocyte infiltration by 41.9%, 64.7%, and 89.4% at doses of 10, 20, and 40 mg/kg, respectively, suggesting significant in vivo anti-inflammatory activity [35].

Hawthorn extract administration caused an anti-inflammatory effect in a carrageenan-induced rat paw edema model, showing 72.4% effectiveness at a dose of 200 mg/kg. In comparison, indomethacin reduces paw edema in rats by 50% at the same dose [35].

- **Antithrombotic effect**

The antithrombotic effect of the methanolic extract was tested in mouse tail models injected with carrageenan, using heparin as a control substance. Extract doses between 200 and 300 mg/kg were the most effective, achieving a significant inhibition of platelet aggregation with high significance [54].

In addition, the ethanolic extract of hawthorn reduced the length of tail thrombosis by 61.5% and 52.5% at 3 and 5 h (at the highest dose of 300 mg/kg), respectively, suggesting that hawthorn could be used as a therapeutic agent against thrombosis [35].

- **Cardioprotective effect**

In rat models, a protective effect against myocardial dysfunction and the occurrence of infarction was observed. In addition, the extracts achieved vasodilator and inotropic effects that affected sodium-potassium (Na^+^/K^+^)-ATPase and favoured the transport of Ca^2+^ ions in cardiomyocytes. In guinea pig ventricular myocytes, a decrease in arrhythmia was observed through a mechanism of action similar to that of class III antiarrhythmics, which reduced cardiomyocyte contraction by activating muscarinic receptors. Certain flavonoids isolated from *C. monogyna*, such as luteolin-7-glucoside, hyperoxide and rutin, produced a cardioprotective effect in the hearts of isolated guinea pigs, at the concentration of 31.25 μg/mL [40,48]. An antiarrhythmic effect was also observed in Wistar rats after the administration of 4 mg/kg of *C. monogyna* ethanolic extract for 60 min by intravenous injection [7]; however, further studies should be performed to confirm if this effect also happens with oral administration of the extracts.

- **Analgesic and gastroprotective effect**

Another ability to highlight is the regulation of the central nervous system, since it depresses its functionality as observed in mice, whose locomotor activity decreased after the administration of *C. monogyna*. For *C. monogyna*, the analgesic and gastroprotective effects were both evaluated following oral administration in rat models. The analgesic effects were observed in mice receiving 10 mg/kg of extract, with activity linked to modulation of the endogenous opioid system. The gastroprotective effects were observed after oral administration of ethanolic extracts at doses up to 200 mg/kg, with protection comparable to ranitidine. Some flavonoids, procyanidins, organic acids, tannins and triterpene derivatives detected in hawthorn extracts have been reported to possess anxiolytic, sedative and analgesic/antinociceptive activities [35].

- **Gastroprotective effect**

The hawthorn berry ethanol extract exhibited significant dose-dependent gastroprotective activity comparable to that of the reference drug ranitidine. Moreover, at the highest tested oral dose (200 mg/kg), the extract produced a stronger protective effect than ranitidine at a dose of 20 mg/kg, but this difference was not statistically significant. Furthermore, several other mechanisms have been proposed to explain their gastroprotective effects, including an increase in mucosal prostaglandin content, a decrease in histamine secretion from mast cells, inhibition of acid secretion, and inhibition of *Helicobacter pylori* growth [48].

In addition, in a rat model of ethanol-induced acute stress ulcer, hawthorn extract demonstrated gastro-protective activity comparable to that of ranitidine, which was used as a positive control [35].

- **Immunomodulatory effect**

Immune responses play a crucial role in defending the body against infections and tumours, with immune cell activation and cytokine secretion serving as the key indicators of these processes. In vivo studies have demonstrated the immunoenhancing properties of an ursolic acid fraction derived from hawthorn, which notably stimulates splenic lymphocyte production and increases the spleen index. Furthermore, research on *C. monogyna* ethyl acetate extract has revealed its ability to enhance the humoral immune response and expand lymphocyte subsets in lymphoid organs, as observed in sheep red blood cell-immunized mice [7].

- **Neuroprotective effect**

Hawthorn’s hydroethanolic extract showed anti-ACE activity, with oleanolic acid as a key component (IC value of 335.00 μg/mL). This indicates that hawthorn could potentially be used to regulate blood pressure through the inhibition of angiotensin-converting enzymes [35].

Hawthorn showed analgesic effects antagonized by naloxone, suggesting opioid receptor-mediated analgesic effects. This finding indicates that hawthorn may have pain-relieving properties similar to those of opioid drugs [35].

### 2.3. Technological Uses of C. monogyna Fruits in Foodstuff

Hawthorn fruits are rich in bioactive compounds and widely used in the food industry for various products, including bakery goods, sweets, meat, alcoholic beverages, fermented products, and functional foods [55,56]. Optimizing extraction conditions is crucial to maximize bioactive compounds, with studies showing ethanol-based solvents and temperatures of 80 °C significantly enhance polyphenol (410%) and anthocyanin (560%) content, improving antioxidant activity for applications in food or cosmetics [57].

Hawthorn pectin has also been investigated for its functional properties. Processing methods such as drying and heat treatment influence pectin’s molecular structure, with hot-air and freeze-dried pectin showing better emulsifying stability [8]. Freeze-thaw cycles enhance juice yield, reduce fruit firmness, and improve sensory qualities by lowering sourness and astringency, making it a valuable technique for improving hawthorn-based food products [58]. Sustainable pectin extraction methods using pure water at room temperature preserve its emulsifying properties and offer an eco-friendly alternative for large-scale production [59].

Hawthorn’s incorporation into bakery products enhances nutritional value and antioxidant properties. Adding hawthorn to whole wheat bread supports digestive and circulatory health, while fruit-enriched cookies exhibit 59% lower acrylamide content and higher antioxidant activity [55,60]. Gluten-free cookies with up to 9% hawthorn powder showed increased phenolic content, antioxidant activity, and improved sensory acceptance [61]. Additionally, hawthorn is used in confectionery to balance its sourness and astringency while enriching its taste and health benefits [55].

In the meat industry, hawthorn extracts effectively reduce lipid oxidation in processed meat products, protecting polyunsaturated fatty acids and inhibiting TBARS formation. Studies confirm their potential to improve the nutritional quality, safety, and sensory properties of meat products, including pork patties and burgers [41,62,63]. Additionally, hawthorn extracts mitigate the pro-oxidative effects of high-oxygen modified atmosphere packaging, preserving meat proteins and lipids while enhancing consumer appeal for clean-label products [64].

Hawthorn also has applications in the beverage industry. Its phenolic compounds serve as flavouring agents in beer and wine, while pectin from wine residues has been successfully incorporated into yogurt and water-kefir production, increasing polyphenol content over 28 days of storage [41,65]. *C. monogyna* plays a key role in food fermentation, improving texture, flavour and shelf life in dairy and meat products. It functions as a natural biopreservative, inhibiting harmful microorganisms while promoting digestive and immune health [6,66].

Lastly, hawthorn-based natural beverages are consumed in countries like Türkiye for their high energy, vitamin, and mineral content, contributing to their popularity as health-promoting drinks [41].

## 3. *Sorbus aria* (L.) Crantz Fruits

*Sorbus aria* (L.) Crantz belongs to the subfamily *Maloideae*, within the family *Rosaceae*, known as whitebeam, and is a deciduous tree or shrub with slow growth, ranging from small to medium size, reaching up to 20 m in height, but usually around 20–25 m. It can be found in all mountainous regions of southern and central Europe, from the Iberian Peninsula and southern Italy to the Balkans, as well as in northern Africa. This species blossoms between May and June, with fruit maturing between September and October. The fruits are subglobose pomes, orange-red in colour, ranging from 10 to 17 × 8–15 mm and containing 1–3 seeds of 5–8 × 2–4.5 mm [13,14,19].

### 3.1. Chemical Composition of S. aria Fruits

*S. aria* fruits display TPC values typically between 900 and 2400 mg GAE/100 g dw [38,67]. *S. aria* fruits are rich in bioactive compounds, notably caffeoylquinic acids (such as chlorogenic acids), flavonoids, and organic acids, which contribute to their potent antioxidant activity (Table 4). Aimone et al. (2024) [67] utilized LC-MS analysis to identify chlorogenic acids as predominant constituents in these extracts.

Petkova et al. (2020) [9] detected four phenolic acids in *S. aria* fruits, with concentrations decreasing in the following order: 2,4-dihydroxybenzoic acid (237.38 ± 0.05 μg/g dw), sinapic acid (179.71 ± 0.18 μg/g dw), p-coumaric acid (145.64 ± 0.20 μg/g dw) and caffeic acid (58.29 ± 0.12 μg/g dw).

Šavikin et al. (2018) [68] reported that *S. aria* extracts contain a wide range of phenolic compounds, predominantly caffeoylquinic acids like neochlorogenic acid (0.18–4.00 mg/g dw) and chlorogenic acid (0.22–2.30 mg/g dw). Additionally, four flavonoids were identified: rutin (138.4–892.0 μg/g dw), isoquercetin (15.5–108.6 μg/g dw), hyperoside (2.3–27.6 μg/g dw) and quercetin (2.1–35.2 μg/g dw).

Olszewska (2008) [69] developed an RP-HPLC method to simultaneously determine four flavonol aglycones in hydrolysed extracts from different parts of *Sorbus* species. In *S. aria* fruits, the total aglycone content was 20 mg/100 g dw, comprising quercetin (9.4 mg/100 g dw), kaempferol (2.4 mg/100 g dw) and isorhamnetin (8.5 mg/100 g dw).

Furthermore, anthocyanin derivatives such as cyanidin-O-hexosyl-O-hexoside, cyanidin-O-hexosyl-pentoside, cyanidin-O-glucoside and cyanidin-O-deoxyhexosyl-pentoside have been identified in *S. aria* epidermis extracts [38].

### 3.2. Biological Activities of S. aria Fruits: In Vitro and In Vivo Studies

The chemical composition and antioxidant activity of the fruits of *S. aria* have not been studied in detail; in fact, scientific literature is scarce. Some data have been reported regarding its content in carotenoids, phenolic acids, flavonoids, and flavonol aglycones [9,38,68,69]. However, information on the detailed chemical composition and antioxidant activity of *S. aria* fruits is limited. In addition, nutrients such as carbohydrates, including pectins and sugars, have not been studied. To the best of our knowledge, no detailed study has been carried out on the physicochemical, nutritional, and antioxidant activities of the fruits of *S. aria* so far [9]. All the information found is detailed in Table 5.

#### 3.2.1. In Vitro Studies

- **Antioxidant effect**

The antioxidant potential of *S. aria* fruits is strongly linked to their high phenolic and flavonoid content. Petkova et al. (2020) [9] reported that total phenol content was twice that of total flavonoids. Various antioxidant capacity assays have been applied, including methods based on single-electron transfer (SET) and hydrogen atom transfer (HAT) mechanisms. The fruits demonstrated antioxidant activity through both mechanisms, with the highest values observed in the CUPRAC assay, followed by DPPH and ABTS radical scavenging tests, while FRAP showed the lowest values. Their significant antioxidant activity suggests their potential as nutraceutical ingredients, particularly due to their low sugar content and high phytochemical concentration, which may contribute to obesity and diabetes management [9].

Olszewska et al. (2009) [70] also assessed antioxidant activity using DPPH, TEAC and FRAP assays, with FRAP yielding the highest results, correlating with phenolic content. Similarly, Özar et al. (2020) [71] investigated the biological activities of *S. aria*, using the Folin-Ciocalteu method for total phenols and CUPRAC for antioxidant analysis, confirming significant antioxidant activity in ethanolic extracts. Šavikin et al. (2018) [68] further established a correlation between total phenolics, proanthocyanidins, and DPPH radical scavenging activity.

Tahirović et al. (2019) [72] confirmed that methanolic extracts exhibited greater antioxidant capacity due to their higher phenolic content, as evidenced by DPPH, TEAC, and FRAP assays. Additionally, Tamayo-Vives et al. (2023) [38] applied the QUENCHER methodology, enabling direct measurement of antioxidant capacity (DPPH, Folin-Ciocalteu, and FRAP) and phenolic composition (Fast-Blue BB, hydroxybenzoic acids, hydroxycinnamic acids, and flavonols) without an extraction process.

- **Antitumor effect**

Antitumor activity was investigated using an ethanol extraction method for white bean fruits. The MTT (bromuro de 3-(3,4-dimetiltiazol-2-il)-2,5-difeniltetrazolio) cell proliferation test was performed to evaluate its effect on prostate cancer. From the MTT cell viability test, the IC50 value was calculated as 25.4 μg/mL for the extract of whitebeam fruit, which is one of the lowest values in the literature [71].

#### 3.2.2. In Vivo Studies

To date, no in vivo studies have been conducted with *S. aria* fruits.

### 3.3. Technological Uses of S. aria Fruits in Food Stuff

There is not much information about the use of white beams in foodstuffs. Bélafi-Bakó et al. (2012) [73] studied the concentration of *S. aria* juice by membrane osmotic distillation, and it was found that approximately 60 total suspended solids were achieved by the process and both the antioxidant capacity, and the total polyphenol content were almost completely preserved, confirming the advantages of the mild membrane process.

In a study by Borcka et al. (2022) [60], other *Sorbus* species were used to bake cookies; in particular, rowan (*Sorbus aucuparia* L.) cookies were one of the most widely accepted and best rated by the panelists. These fruit-enriched cookies had significantly higher antioxidative properties than the control cookies, with high polyphenol content, which explains why the acrylamide content of the fruit-enriched cookies was significantly lower. In this regard, cookies enriched with wild fruits constitute a promising novel snack food.

These fruits contain different nutrients that are of interest to the food industry for the formulation of new products. Carbohydrates are the main constituents of the fruit, reaching 38.54 g/100 g of fresh weight since soluble sugars accounted for 18%. Sucrose was not detected in the aqueous extract, whereas fructose and glucose were the only monosaccharides present in the extract (2.73 and 3.63 g/100 g fresh fruit, respectively). The total sugar content was 6.36 g/100 g fresh weight. Therefore, the taste of *S. aria* depends on its free sugars (glucose and fructose) [9].

Another valuable component detected in the fruit of *S. aria* was uronic acids, which represent the amount of acidic polysaccharides such as pectin (1.77 0.02 g/100 g fresh weight). They represent 5% of the total carbohydrates in fruits [9].

Pectin is a valuable soluble fibre that is widely used in the food industry because of its thickening and gelling properties. Therefore, these fruits represent a natural low-calorie source of dietary fibre and could be successfully used for the preparation of jams, jellies, fillings, and jams [9].

Finally, different natural pigments were identified in the *S. aria* fruits. Total chlorophylls were 5.12 μg/g fresh weight, while total carotenoids were 3 times higher (16.91 μg/g fresh weight). Chlorophylls a and b were also detected in the fruit of *S. aria* with mean values of 0.61 μg/g dry weight for chlorophyll a and 0.43 μg/g dry weight for chlorophyll b. The total carotenoid content was higher than the chlorophyll content. β-carotene and lycopene were the main carotenoids detected, with a total content of 69% of the total carotenoids. In addition, lycopene is one of the main carotenoid constituents of *S. aria* fruits. Finally, monomeric anthocyanins were not detected in the fruits of *S. aria* [9].

## 4. *Prunus spinosa* L. Fruits

*Prunus spinosa* L., commonly known as blackthorn, belongs to the subfamily *Amygdaloideae*, within the family *Rosaceae*, and is naturally found in Europe, northwest Africa, and western Asia, where it has been used therapeutically for many years. It is a small, evergreen, spiny scrub that can reach up to 1–2.5 m in height and flowers between April and May. The fruits of the blackthorn are 10–15 mm, subglobose or ovoid drupes with a black-blueish color and acidic-astringent taste, and they ripen in autumn [13,14,19].

### 4.1. Chemical Composition of P. spinosa Fruits

The fruits of *P. spinosa* contain numerous compounds responsible for their bioactive properties; these compounds are detailed in Table 6. Organic acids in *P. spinosa* were analysed using HPLC, with malic, citric, quinic, shikimic and fumaric acids identified as the main components [74]. Celik et al. (2017) [75] reported citric, ascorbic, succinic, fumaric and malic acids in descending order, while Morales et al. (2013) [21] quantified malic (912.71 mg/100 g dw), oxalic (299.15 mg/100 g dw) and citric acid (170.13 mg/100 g dw).

The phytochemical composition of *P. spinosa* is highly influenced by solvents and extraction methods. *P. spinosa* shows a TPC range from 1100 to 3200 mg GAE/100 g dw, particularly when using optimized extraction of epicarp and enriched fractions [11] as noted in a review by Bei et al. (2024) [76]. Magiera et al. (2022) [12] identified neochlorogenic acid (4962 mg/100 g dw) and rutin (293 mg/100 g dw) across all extracts, with the highest levels obtained using ethyl acetate and n-butanol fractions. Mikulic-Petkovsek et al. (2016) [74] reported cinnamic acids as predominant, followed by flavonols, flavanols and flavones, with 3-caffeoylquinic acid, quercetin pentoside 3, procyanidin dimer 2, and apigenin pentoside as major compounds. Celik et al. (2017) [75] detected rutin along with vanillic, gallic, syringic and ferulic acids.

Negrean et al. (2023) [77] found quercetin and its glycosides (e.g., rutin) in higher concentrations in the peel, alongside caffeic acids, catechin, epicatechin, kaempferol, gallic, chlorogenic, syringic, vanillic, ferulic and p-coumaric acids. Marcetic et al. (2022) [78] identified caffeoylquinic acid and quercetin as dominant in *P. spinosa*, with neochlorogenic and chlorogenic acids as the primary phenolic acids. Najgebauer-Lejko et al. (2021) [79] found myricetin as the only flavonoid in *P. spinosa* puree, while chlorogenic (144.98 mg/100 g dw) and caffeic acids (100.39 mg/100 g dw) were the most abundant phenolics. Stoenescu et al. (2022) [34] detected only gallic, neochlorogenic, and caffeic acids.

Marcetic et al. (2022) [78] employed HPLC-DAD-ESI-MS to analyse methanol, water, and hydroethanolic extracts of ripe *P. spinosa* fruits, finding higher concentrations of caffeoylquinic acid 1, feruloylquinic acid, and quercetin derivatives in methanol extracts. Sabatini et al. (2020) [80] separated and quantified flavanols, hydroxycinnamic acids and benzoic acids in prune extracts via HPLC-DAD, including caffeic, coumaric, chlorogenic, gallic, syringic, vanillic acids and (+)-catechin. However, neochlorogenic acid was absent in *P. spinosa* fruit extracts.

Anthocyanins in *P. spinosa* include cyanidin 3-O-rutinoside and peonidin 3-O-rutinoside [81,82]. González-de-Peredo et al. (2020) [83] identified cyanidin 3-O-glucoside, cyanidin 3-O-rutinoside, peonidin 3-O-glucoside and peonidin 3-O-rutinoside [12,77,80,84,85]. Guimarães et al. (2013) [86] found additional anthocyanins such as cyanidin 3-O-pentoside, peonidin 3-O-pentoside, cyanidin 3-O-acetylglucoside and peonidin 3-O-acetylglucoside. Bei et al. (2024) [76] expanded the list by identifying peonidin 3-O-rhamnoside and pelargonidin 3-O-glucoside, while Nistor et al. (2023) [87] reported delphinidin 3-O-glucoside.

### 4.2. Biological Activities of P. spinosa Fruits: In Vitro and In Vivo Studies

As mentioned before, the blackthorn fruits contain various phenolic compounds, with anthocyanins being the most abundant phenolic component. In *P. spinosa* extract, cyanins account for approximately 58% of phenolic compounds, followed by hydroxycinnamic acids, which make up approximately 22% [10]. Those compounds are responsible for the biological activities of *P. spinosa* (Table 7).

#### 4.2.1. In Vitro Studies

- **Antioxidant effect**

Several studies confirm the strong antioxidant activity of *P. spinosa* fruits. Magiera et al. (2022) [12] demonstrated that methanolic extracts neutralized reactive oxygen species (ROS) due to their high content of phenolic compounds, including flavonoids, anthocyanins, phenolic acids and proanthocyanins. The extract also protected against lipid peroxidation by reducing TBARS levels and exhibited antioxidant capacity in the FRAP assay.

Pozzo et al. (2019) [10] found that *P. spinosa* aqueous extracts significantly enhanced cellular antioxidant activity (AAC) in erythrocytes. The extract inhibited AAPH-induced haemolysis, likely due to its high gallic acid, rutin and quercetin content. Similarly, Coppari et al. (2021) [11] confirmed the dose-dependent antioxidant effects of *P. spinosa* polyphenols via the DPPH assay. Sabatini et al. (2020) [80] observed concentration-dependent antioxidant activity in ethanolic extracts, attributing this effect to anthocyanins, flavonols, cinnamic acids and benzoic acids.

Marčetić et al. (2022) [78] compared *P. spinosa* methanolic extracts from two localities, finding significant differences in antioxidant capacity (DPPH, ABTS and FRAP assays), with higher activity linked to greater phenolic content. Other compounds, including organic acids, vitamins (ascorbic acid, tocopherols) and carotenoids, also contribute to the fruit’s antioxidant potential. Backes et al. (2020) [82] found that *P. spinosa* extract provided greater cellular protection against oxidation than fig peel extract, as assessed by TBARS and OxHLIA assays, although Trolox showed the lowest antioxidant activity.

Oxidative stress is crucial in diabetes complications, particularly in the cardiovascular system. Peroxynitrite (ONOO^−^), a highly toxic ROS/RNS generated in hyperglycaemia, damages biomolecules [12]. To assess *P. spinosa*’s protective effects, an experimental model of human blood plasma under oxidative stress was developed. The extract enhanced the non-enzymatic antioxidant capacity of plasma and acted as a major plasma protective agent due to its high flavonoid and phenolic acid content [12].

Additionally, *P. spinosa* extracts inhibited protein glycation, as shown in bovine serum albumin (BSA) assays. Advanced Glycation End Products (AGEs), formed by oxidation and glycation of biomolecules, were significantly reduced by both fresh and dried *P. spinosa* extracts, preventing oxidative damage in vivo [12].

- **Antimicrobial effect**

Several studies confirm the antimicrobial activity of *P. spinosa* fruit extracts against Gram-negative and Gram-positive bacteria. Pozzo et al. (2019) [10] found that even at 0.25 mg/mL, the extract inhibited more than 50% of *Escherichia coli*, *Salmonella typhimurium*, *Enterobacter aerogenes*, *Enterococcus faecalis* and *Staphylococcus aureus* growth. The antimicrobial action is attributed to phenolic compounds, particularly flavonoids and phenolic acids, which damage bacterial membranes and suppress virulence factors.

Using MIC and MBC/MFC values, Sabatini et al. (2020) [80] confirmed that *P. spinosa* extracts exhibited broad antibacterial activity but had limited antifungal effects. Similarly, Marčetić et al. (2022) [78] reported that extracts effectively inhibited pathogenic bacteria and yeast, particularly *Escherichia*, *Salmonella* and *Staphylococcus* species, suggesting flavonoids and phenolic acids as key antimicrobial agents.

Additionally, *P. spinosa* extracts were active against antibiotic-resistant bacteria, though their effectiveness varied depending on bacterial species [82].

A novel antimicrobial approach using photodynamic inactivation was explored by Olszewska et al. (2024) [88], combining *P. spinosa* fruit peel extracts with blue light treatment to target *Listeria monocytogenes*. Rich in quercetin and rutin, the extracts enhanced bacterial reduction by up to four logarithmic units after 15 min of illumination at 58.5 J/cm^2^. Flow cytometry revealed extensive membrane damage in over 90% of bacterial cells, highlighting blackthorn fruit peel as a natural photosensitizer for food safety applications.

- **Anti-inflammatory effect**

The potential of *P. spinosa* fruit extract to reduce inflammation in laboratory cell models has been widely investigated. Although the interaction between the extract and a protein called TLR4 is not exactly known, it has been found that the extract can internally regulate miR-126, which reduces the inflammatory response [11,80].

TLR4 is a receptor that is part of the immune system and recognizes lipopolysaccharides from gram-negative bacteria. Its activation triggers inflammatory and defensive responses to fight infection. TLR4 interacts with and stimulates IRAK1, a protein that plays an important role in immune system signalling within cells, activating the transcription factor NFkB to express miR-146a, IL-6, and cell adhesion molecules (ICAM-1 and VCAM-1) [11,80].

miR-146a is a small RNA molecule that plays a crucial role in the regulation of inflammation. ICAM-1 and VCAM-1 are cell surface proteins that are involved in the inflammatory response. ICAM-1 and VCAM-1 levels may increase during inflammation, contributing to the adhesion and migration of inflammatory cells. IL-6 is a protein that acts as a chemical signal in the body’s immune system, is produced in response to inflammation, and plays an important role in the regulation of the immune response, promoting inflammation, and triggering defence responses [11,80].

When cells are treated with *P. spinosa* extract, TLR4 signalling can be blocked by a negative feedback loop, where miR-146a targets IRAK-1, leading to a decrease in the expression of IL-6 and cell adhesion molecules (ICAM-1, VCAM-1) [11,80].

- **Hypoglycemic effect**

Inhibition of the enzymes α-amylase and α-glucosidase may reduce blood glucose absorption and suppress hyperglycaemia after meals. Plant extracts such as those from *Prunus* fruits, including blackthorns, have shown antidiabetic properties, although the type of extract used and the genotype of the sloes may affect the inhibition of these enzymes. The hydroalcoholic extract of *P. spinosa* has demonstrated a greater ability to inhibit α-amylase and α-glucosidase than the water extract, possibly because of its high flavonoid content [12,78].

Anthocyanins and quercetin glycosides present in wild sloes show inhibitory activity against these enzymes. Phenolic compounds contributed mainly to the observed effects, and extracts of fresh fruits of *P. spinosa* had more relevant results than those obtained from dried blackthorns. These findings support the therapeutic application of *P. spinosa* fruit in diabetes, as recommended in traditional medicine [12,78].

Moreover, cyanidins, such as cyanidin 3-O-gloside and cyanidin 3-O-rutinoside, have a healthy effect against diabetes and associated metabolic diseases because of the reduction of diabetes, obesity, and postprandial glucose by inhibition of pancreatic α-amylase and intestinal α-glucosidase, as mentioned before, and the diminution of glucose transport in the small intestine and inhibition of glucose uptake in colorectal adenocarcinoma epithelial cells [76].

- **Effect on cellular senescence**

In the study by Coppari et al. 2021 [11], *P. spinosa* extract appeared to have an important “qualitative” effect on cell differentiation, at least at certain concentrations. These results suggest that the antioxidant and anti-inflammatory effects of the ethanolic extract of *P. spinosa* fruits may allow older cells to behave as if they are younger, coping with the pro-inflammatory phenotype associated with cellular senescence. In fact, aged cells treated with the extract at the same rate as controls behaved as if they were “biologically” younger [11].

- **Neuroprotective effect**

In addition to catalysing the hydrolysis of the neurotransmitter acetylcholine, the enzyme acetylcholinesterase (AChE) has many different functions, including modulation of oxidative stress, inflammatory and apoptotic responses, and morphogenic and adhesion functions. In addition to their antioxidant and anti-inflammatory properties, there is evidence that bioactive compounds from several *Prunus* species exert neuroprotective effects by inhibiting AChE activity. Extracts with higher content of phenolic acids and flavonoids showed approximately four times more AChE inhibitory activity than the positive control, galantamine [78].

Bei et al. (2024) [76] highlighted the neurodegenerative effects of flavonoids such as quercetin and rutin, the main outcomes of which are AChE and monoamine oxidase inhibition, diminution of peroxyl radical capture and oxidation, neurotrophic action, and maintenance of the physiological functions of vital organs.

The neuroprotective potential against Alzheimer’s disease was assessed, revealing that blackthorn skin extracts (1–10 μg/g) inhibited the aggregation and protected against oxidative stress in SH-SY5Y cell lines. A multivariate analysis correlated the phenolic composition with bioactivities, emphasizing the potential use of these residues as neuroprotective agents in the nutraceutical industry [89].

- **UV photoprotective effect**

Tyrosinase is involved in the oxidation of tyrosine to melanin, and its overproduction is associated with hyperpigmentation, skin photocarcinogenesis, and neurodegenerative processes. Previous data on the tyrosinase inhibitory activity of *P. spinosa* fruit are limited. Considerable tyrosinase inhibition capacity was observed for 45% propylene glycol extract (*v*/*v*), suggesting that it could be attributed to its higher antioxidant activity and number of total phenols than other types of extracts (water, 70% (*v*/*v*) ethanol, and methanol). Other authors have proposed that the presence of ellagic and vanillic acids and the chelation capacity of metals could be important factors in determining the anti-tyrosinase properties of sloe fruit extracts; however, they exerted less activity than kojic acid [78]. This result is a starting point to investigate the possibility of developing such an effect through oral ingestion, as a contribution to oxidative balance in different parts of the body, including the skin.

- **Antitumor effect**

The methyl thiazole tetrazolium (MTT) test is a colorimetric assay for measuring cellular metabolic activity and is particularly suitable for the evaluation of inhibition of cell viability, as it can quantify drug-induced mitochondrial damage. The study by Condello et al. 2019 [90] evaluated the effects of a food supplement based on *P. spinosa* drupe extract combined with other ingredients, such as B3, B6, B1, B2, B12 vitamins (less than 15% with respect to nutrient reference values) and amino acids, but the presence of a significant amount of phenolic acids (39.95 mg/100 g), of flavonols (66.91 mg/100 g) and anthocyanins (100.81 μg/100 g) from *P. spinosa* drupe extract makes it particularly interesting. This product induces apoptosis, which is the main mechanism of programmed cell death. In comparison with 5-fluorouracil treatment, the food supplement mentioned had a cytotoxic effect due to apoptotic induction. This can be observed in a morphological analysis due to the modified mitochondrial membrane potential, with an increase of 20% depolarized mitochondria. In contrast, quantitative data from 3D line analysis confirmed the results obtained from morphological analysis, indicating that treatment of colon carcinoma cells with the natural antioxidant compound may contribute to cytotoxicity in a combination of the *P. spinosa*-based food supplement with the chemotherapeutic agent [90]. Further research is required to confirm these findings.

Phenolic acids have been demonstrated to have cytotoxic activity in some cancer cell lines, inducing in vitro endogenous antioxidant mechanisms and modulating Nrf2 transcription factors, a regulator of cellular resistance to oxidative damage, and decreasing the disruption of the pro-oxidant/antioxidant balance, with a key role in some cancers [76].

- **Cardioprotective effect**

Many bioactive compounds, such as flavonoids, proanthocyanidins, and anthocyanidins, including cyanidin 3-O-rutinoside and cyanidin 3-O-glycoside, have positive cardiovascular effects. Flavonoids inhibit pro-inflammatory enzymes, have antiatherosclerotic and antithrombotic properties, and modulate lipid metabolism by normalizing the LDL/HDL ratio. Moreover, flavonoids improve capillary permeability and endothelial function with vasodilatory effects, increasing the release of nitric oxide, uncoupling the endothelial nitric oxide synthesis, and decreasing oxidative DNA damage [76].

Furthermore, proanthocyanidins have cardioprotective effects that modulate lipid metabolism, increase the antioxidant capacity of plasma, improve vascular function, and decrease platelet activity [76].

Regarding anthocyanins, cyanidin 3-O-rutinoside is related to cardiovascular effects by improving lipid-decreasing mechanisms, antioxidant activity on ROS, antiglycation activity, inhibition of lipolytic digestive enzymes, lipid absorption processes, cholesterol mycelial formation linked to primary and secondary bile acid, pancreatic lipase, pancreatic cholesterol esterase, and cholesterol mycelial absorption in the proximal jejunum. Cyanidin 3-O-glycoside is related to increased tissue tolerance to ischemic injury, reducing the risk of cardiovascular diseases and hypertension, having the capacity to scavenge ROS and decrease oxidative stress, thereby enhancing inflammatory responses [76].

#### 4.2.2. In Vivo Studies

- **Antioxidant Effect**

Supplementation with *P. spinosa* fruit has been shown to have antioxidant properties in vivo, reducing oxidative stress in the liver and brain. This was attributed to the presence of various polyphenols, such as rutin, 4-hydroxybenzoic acid, gallic acid, and quercetin. Regularly consuming these fruits could increase the circulation of these bioactive compounds, thereby improving the endogenous antioxidant system and protecting tissues against damage caused by a high-fat diet and hyperglycaemia. These fruits can be used in the production of functional foods and nutraceuticals as well as in food processing [10].

The nematode *Caenorhabditis elegans* is a widely used model for studying the effects of different natural and synthetic compounds, including polyphenols, on aging and longevity. Changes in gene expression and the regulation of microRNAs play important roles in these processes. For example, overexpression of miR-124 in *C. elegans* increases life expectancy, whereas loss of miR-124 accelerates aging. In addition, miR-39 has been shown to be involved in specific age-related changes [11].

A study on *C. elegans* worms to validate the antioxidant effects of *P. spinosa* fruits in vivo was conducted. The resistance of the worms to oxidative stress induced by hydrogen peroxide (H_2_O_2_) was tested. Worms fed *P. spinosa* extract showed significant protection against the harmful effects of peroxide compared to the control group. Therefore, *P. spinosa* fruits have antioxidant properties, can improve the endogenous antioxidant system, and protect against oxidative stress. In addition, its beneficial effects on aging have been demonstrated using a *C. elegans* model [11].

- **Longevity study**

Life expectancy analysis was performed using wild *C. elegans* worms exposed to different concentrations of *P. spinosa* extract throughout the lifespan. Bioavailability plays a key role in assessing health effects in living organisms. Therefore, the concentrations used in in vivo assays are different and (often) higher than those tested in vitro. The bioavailability of *P. spinosa* fruits is unknown; therefore, from a concentration (40 μg/mL) that had been shown to be effective in in vitro or in vivo experiments, it was decided to also test ten times lower (4 μg/mL) and ten times higher (400 μg/mL) in *C. elegans*. The survival curves of the treatment versus control groups (ethanol) showed that *C. elegans* worms treated with different concentrations (4, 40 and 400 μg/mL) of *P. spinosa* ethanolic extract showed no differences between themselves and the control until the first 10 days. Worms treated with the highest concentration (400 μg/mL) showed a significantly extended shelf life compared with the other experimental conditions [11].

- **MicroRNA modulation**

It has already been shown that overexpression of miR-124 prolongs the lifespan of *C. elegans*, suggesting that miR-124 may be involved in the regulation of longevity in *C. elegans*, and that the loss of miR-124 in *C. elegans* increases the formation of reactive oxygen species (ROS) and the accumulation of markers of aging, resulting in a reduction in service life. In addition, miR-39 has been shown to be involved in modulating strong age-specific changes. On this basis, Coppari et al. (2021) [11] analysed the expression of miR-124 and miR-39 in *C. elegans* treated with *P. spinosa* fruits. The study showed that the expression of miR-124 increased after treatment with *P. spinosa*, whereas miR-39 expression decreased. Both results suggest a possible positive effect of *P. spinosa* ethanolic extract on the shelf life of *C. elegans* by modulating the expression levels of miR-124 and miR-39. Overall, these findings reveal that *P. spinosa* fruit extract may have antioxidant effects in vivo, extending their life, promoting healthy living, and improving the stress resistance of *C. elegans* [11].

- **Antitumor effect**

Condello et al. (2019) [90] evaluated the effects of *P. spinosa* ethanolic extract-based supplementation on colon carcinoma tumour growth in immunodeficient mice. Tumours rapidly progressed in the control group, leading to morbidity and death within 25 days. In contrast, mice receiving *P. spinosa* drupe extract-based supplementation (containing phenolic acids, flavonols, and anthocyanins) exhibited slower tumour progression, with no significant differences between low and high doses.

A separate toxicological study assessed the safety of the supplement at 0.05 and 0.15 mg/mL doses, administered orally for one month. No toxicity, weight loss, or organ pathology was observed. Histological analysis revealed significantly lower tumour necrosis in treated mice—approximately 10% or less in supplemented groups compared to 30% in controls, with higher doses yielding greater necrosis reduction [90].

In summary, *P. spinosa* extract-based supplementation effectively slowed tumour growth and reduced necrosis without harming normal colon tissue. These findings suggest its potential integration into multi-agent cancer treatment protocols, though further research is required to clarify its antitumor mechanisms, chemosensitizing properties, and immune modulation potential [90].

- **Gastroprotective effect**

The anti-ulcer properties of *P. spinosa* were investigated because of its antioxidant, anti-inflammatory, and wound-healing effects. Ethanol extracts of *P. spinosa* fruits were administered to Wistar albino rats at doses of 100 mg/kg and 200 mg/kg using an indomethacin-induced gastric ulcer model. Ulcer areas on the stomach surface were examined macroscopically, and the tissues were analysed histopathologically and biochemically. Higher levels of TNF-α, IL-6, IL-1β, IL-8, and NF-kB were observed in the gastric ulcer group than in the extract-treated groups. No differences in VEGF levels were detected among the groups, while significant differences in PGE2 levels were identified between the lansoprazole and high-dose extract groups. Reduced neutrophilic infiltration in the gastric mucosa was histopathologically observed in the extract-treated groups. Ascorbic acid, homoprotocatechuic acid, and genistein were the main compounds in the extract. Protection of the gastric mucosa was demonstrated through the modulation of inflammation and PGE2 pathway by *P. spinosa*, with its effects shown to be dose-dependent and comparable to or more effective than the reference substance [91].

### 4.3. Technological Uses of P. spinosa Fruit in Food Stuff

Blackthorn has gained attention for its potential applications in functional foods, fermented products, and natural colorants. Fraternale et al. (2009) [92] highlighted its use in dietary supplements, while Marcetic et al. (2022) [78] investigated its prebiotic effects, particularly in promoting Saccharomyces boulardii growth. Anthocyanins in blackthorn were shown to inhibit harmful bacteria through gene regulation and metabolic changes [93].

Blackthorn enhances probiotic yogurt by increasing polyphenol and anthocyanin content, improving antioxidant capacity, and serving as a natural colorant and dietary fibre source [79]. Encapsulated blackthorn extracts enable controlled bioactive compound release in yogurt, enhancing bioavailability [77,94].

*P. spinosa* powder enrichment in rice flour cakes improved nutritional value by increasing protein, moisture, and antioxidant content while reducing glycaemic index [95]. However, higher levels affected texture by reducing volume and increasing hardness over storage.

The industrial processing of blackthorn vinegar using UV-C light and ultrasonication preserved bioactive compounds, phenolics, and volatile compounds better than pasteurization, enhancing its anticarcinogenic activity [96].

Mandic et al. (2018) [97] demonstrated that blackthorn extract improved vacuum-packed sausages by reducing microbial growth and extending shelf life. It also showed potential for enhancing antioxidant function when added to sausage fillings [77].

Blackthorn is a valuable source of natural colorants. Anthocyanin-rich extracts, obtained via ultrasound and heat-assisted extraction, were tested in doughnut icing, initially providing a dark purple tone that faded over time, yet retaining high antioxidant and antimicrobial properties [77,81,82].

Bei et al. (2024) [76] reviewed blackthorn’s broad functional applications, highlighting its use in ice cream, isotonic drinks, kombucha, herbal teas and alcoholic beverages. These fruits are a sustainable source for syrups, juices, wines, and tinctures, reinforcing their nutritional, sensory, and functional benefits.

## 5. Conclusions and Future Perspectives

Many advances have been made in the last few decades regarding the bioactivity of different fruits and fruit extracts. The fruits of *C. monogyna*, *S. aria* and *P. spinosa* have been shown to contain phenolic acids (such as sinapic, chlorogenic, caffeic, caffeoylquinic or coumaric acids), as well as flavonoids (quercetin, kaempferol, myricetin or rutin) and anthocyanins (such as cyanidin, malvidin, peonidin, petunidin or delphinidin derivatives), with differences in their specific profile and contents. Phenolic compounds are generally extracted with polar solvents (ethanol, methanol, water), and their stability depends on storage temperature, pH, and protection from light [30,38].

These compounds are related to their biological activities, as demonstrated by in vitro and/or in vivo assays. *C. monogyna* fruits have shown antioxidant, antitumor, anticoagulant, hypoglycaemic, and UV photoprotective activities in vitro, moderate antimicrobial activity, and cardioprotective effects in both in vitro and in vivo assays. The antioxidant and antitumor activities of *S. aria* fruits have been reported through in vitro assays; however, no in vivo studies have been conducted on these fruits. The fruits of *P. spinosa* are promising for their antioxidant, anti-inflammatory, hypoglycaemic, neuroprotective, and UV photoprotective in vitro properties, as well as antimicrobial activity against several microorganisms. In vivo studies using *C. elegans* have corroborated some of these effects, mainly antioxidant properties.

Regarding the feasibility of incorporating *C. monogyna*, *S. aria*, and *P. spinosa* into modern diets, such as food, food ingredients, and dietary supplements, the promising bioactivities of these fruits, demonstrated primarily through in vitro studies, suggest their potential as valuable components in functional foods and nutraceuticals. However, their successful inclusion requires addressing practical challenges such as optimizing preservation methods, enhancing palatability, and ensuring safety for widespread consumption, including allergenic potential. This integration not only aligns with consumer demand for natural and health-promoting products but also provides an opportunity to revitalize traditional dietary practices with scientific support.

With this panorama, it is essential to promote further research, particularly in vivo and human intervention studies. These studies should evaluate physiological effects, including the measurement of function markers related to the effect proposed, and the dose–response relationship, which is crucial to have a high level of scientific evidence to substantiate potential health claims for these ingredients. A further step would be clinical trials focusing on pharmacological activities. Such efforts are a critical milestone in enabling their practical application as active compounds in the food sector.

## Figures and Tables

**Table 1:** Traditional uses of fruits of *Crataegus monogyna* Jacq., *Sorbus aria* (L.) Crantz and *Prunus spinosa* L.

Fruits	Uses	Properties	Conditions Treated	References
***C. monogyna***	Tinctures, infusions, or liquid extractions	Cardiotonic, hypotensive, antidiarrheal, hepatoprotective, anxiolytic, soothing and sedative	Improves blood circulation, hepatitis processes, bronchitis and respiratory infections, insomnia	[1,6,7,8]
***S. aria***	Fresh, dried, or processed as jam, preserves, syrup, vinegar, brandy, liqueurs, and fruit wine or added to bread flour (German name “Mehlbeere = Flour Berry”)	Food ingredients, and as a traditional diuretic, anti-inflammatory, antidiarrheal (nuts), vasodilators and for its vitamin content	Improves diuresis, inflammatory processes, diarrheal processes, and blood circulation. Vitamin supplement for micronutrients	[9]
***P. spinosa***	Traditional production of jams and concoctions such as juices, wines, teas, and spirits	Ingredients in the food industry and treatment of various diseases in phytotherapy, such as cough treatment, diuretic, laxative, antispasmodic and anti-inflammatory.	Improves diuresis, spasmolytic, antimicrobial and antioxidant activity	[10,11]

**Table 4:** Organic acids and phenolic compounds found in the fruits of *Sorbus aria* (L.) Crantz.

Compound Families		Compound	Reference
Organic acids		Sorbic acid	[67]
		Methyl esters of sorbic acid	
Phenolic acids	Hydroxycinnamic acids	Sinapic acid	
		p-coumaric acid	
		Caffeic acid	[9,67,68]
		Chlorogenic acid	
		Neochlorogenic acid	
	Hydroxybenzoic acids	2,4-dihydrohybenzoic acid	[9]
Flavonoids	Flavonols	Quercetin	
		Rutin	
		Isoquercetin	[67,68,69]
		Hyperoside	
		Kaempferol	
		Isorhamnetin	
	Anthocyanins	Cyanidin-O-hexosyl-O-hexoxide	
		Cyanidin-O-hexosyl-pentoxide	
		Cyanidin-O-glucoside	[38]
		Cyanidin-O-deoxyhexosyl-pentoxide	
		Cyanidin derivate	

**Table 5:** In vitro studies on bioactive compounds in *S. aria*. fruits.

Effects	Study	Solvent	Compounds Responsible for Activity	References
**In vitro studies**				
Antioxidant	Radical scavenging capacity (DPPH, ABTS+), ferric reducing antioxidant power (FRAP), cupric reducing antioxidant capacity (CUPRAC).	Ethanol	Phenolic compounds, flavonoids, pigments	[9]
	DPPH test, TEAC assay, and FRAP assay.	Methanol 70%	Phenolic compounds and flavonoids	[70]
	Folin-Ciocalteu method and CUPRAC method.	Ethanol	Phenolic compounds and flavonoids	[71]
	Folin-Ciocalteu method and DPPH test.	Ethanol 50%	Phenolic compounds and flavonoids	[68]
	DPPH, TEAC and FRAP assays.	Methanol 80%	Phenolic compounds and flavonoids	[72]
	DPPH, Folin-Ciocalteu, FRAP, Fast Blue BB.	Not determined	Phenolic compounds and flavonoids	[38]
Antitumor	MTT cell proliferation test to evaluate the anticancer effect on prostate cancer.	Ethanol	Phenolic compounds and flavonoids	[71]

**Table 6:** Organic acids and phenolic compounds found in the fruits of *Prunus spinosa* L.

Compound Families		Compound	Reference
Organic acids		Citric	
		Malic	
		Quinic	
		Shikimic	
		Fumaric	[21,74,75]
		Succinic	
		Ascorbic	
		Dehydroascorbic	
		Oxalic	
Phenolic acids	Hydroxycinnamic acids	3-p-Coumaroylquinic acid	
		4-p-Coumaroylquinic acid 1	
		Caffeic acid hexoside 1	
		Caffeic acid hexoside 3	
		p-Coumaric acid hexoside 1	
		3-Caffeoylquinic acid (neochlorogenic acid)	
		4-Caffeoylquinic acid (cryptochlorogenic acid)	[12,34,74,75,76,77,78,79,80]
		5-Caffeoylquinic acid 1 (chlorogenic acid)	
		3-Feruloylquinic acid	
		Caffeoylshikimic acid derivative	
		Syringic acid	
		Ferulic acid	
		Feruloylquinic acid	
	Hydroxybenzoic acids	Vanillic acid	
		Vanilloyl malate hexoside	
		Protocatechuic acid 4-O-hexoside	[12,34,74,75,76,77,78,79,80]
		Protocatechuic acid	
		p-Hydroxybenzoic acid	
		Gallic acid	
Flavonoids	Flavan-3-ols	(+)-catechin	[80]
		Epicatechin	
	Flavanols	Procyanidin dimer 1	[74]
		Procyanidin dimer 2	
		Procyanidin trimer 1	
	Flavones	Apigenin pentoside	[74]
	Flavonols	Quercetin triglycoside	
		Quercetin acetyl hexoside	
		Quercetin deoxyhexoside	
		Quercetin deoxyhexoside-hexoside 1	
		Quercetin deoxyhexoside-hexoside 2	
		Quercetin acetyl rutinoside	
		Quercetin hexoside 1	
		Quercetin hexoside 2	
		Quercetin hexosyl pentoside 1	
		Quercetin hexosyl pentoside 2	
		Quercetin hexosyl rhamnoside	
		Quercetin-3-xyloside	[12,34,74,75,76,77,78,79,80]
		Quercetin pentoside 1	
		Quercetin pentoside 2	
		Quercetin pentoside 3	
		Quercetin rhamnosyl hexoside	
		Querectin-3-galactoside	
		Quercetin-3-glucoside (isoquercetin)	
		Quercetin-3-rhamnoside	
		Quercetin-3-rutinoside	
		Methylquercetin pentoside 1	
		Methylquercetin deoxyhexoside-hexoside	
		Isorhamnetin hexoside	
		Kaempferol pentoside hexoside	
		Kaempferol rhamnosyl hexoside 1	
		Kaempferol rhamnosyl hexoside 2	
		Kaempferol pentoside	
		Rutin	
		Myricetin	
	Anthocyanins	Cyanidin 3-O-glucoside	
		Cyanidin 3-O-rutinoside	
		Peonidin 3-O-glucoside	
		Peonidin 3-O-rutinoside	
		Cyanidin 3-O-pentoside	[12,76,77,80,81,82,83,84,85,86,87]
		Peonidin 3-O-pentoside	
		Peonidin 3-O-rhamnoside	
		Cyanidin 3-O-acetylglucoside	
		Peonidin 3-O-acetylglucoside	
		Delphinidin 3-O-glucoside	
		Pelargonidin 3-O-glucoside	

**Table 7:** In vitro and in vivo studies on bioactive compounds in *P. spinosa* fruits.

Effects	Study	Solvent	Compounds Responsible for Activity	References
In vitro studies				
Antioxidant	Antioxidant capacity of extracts towards several ROS/RNS of physiological importance (O_2_^•−^, HO^•^, H_2_O_2_, NO^•^, HOCl).	Methanol 75%	Polyphenolic components, such as flavonoids, anthocyanins, and phenolic acids derivatives and proanthocyanins	[12]
	Cellular antioxidant activity (AAC) in human erythrocytes, inhibition of dihydrochloride-induced oxidative haemolysis (AAPH) in human erythrocytes.	Distilled water	Phenolic compounds (gallic acid, rutin, quercetin)	[10]
	Radical scavenging effect of DPPH.	Ethanol 70% acidified with HCl	Phenolic compound	[11]
	Radical Scavenging Capacity (DPPH).	Ethanol 70%	Phenolic compounds	[80]
	DPPH and ABTS radical scavenging effect, ferric reducing antioxidant power (FRAP), inhibition of β-carotene bleaching and inhibition of lipid peroxidation of brain cells.	Methanol	Phenolic compounds, flavonoids and anthocyanins	[78]
	Thiobarbituric acid reactive substances assay (TBARS), inhibition of oxidative haemolysis (OxHLIA).	Ethanol 50%	Anthocyanins (cyanidin-3-rutinoside and peonidin-3-rutinoside)	[82]
	To verify the preventive characteristics of extracts against peroxidation and nitration of human plasma components, and their impact on the non-enzymatic antioxidant capacity of plasma under conditions of oxidative stress.	Methanol 75%	Polyphenolic components, such as flavonoids, anthocyanins, and phenolic acid derivatives and proanthocyanins	[12]
	Evaluation of the anti-glycation properties of extracts (the impact on the formation of AGEs).	Methanol 75%	Polyphenolic components, such as flavonoids, anthocyanins, and phenolic acid derivatives and proanthocyanins	[12]
Antimicrobial	Inhibition of Gram-negative bacteria *Escherichia coli*, *Salmonella typhimurium* and inhibition of the growth of *Enterobacter aerogenes*. Inhibition of Gram-positive bacteria *Enterococcus faecalis* and inhibition of the growth of *Staphylococcus aureus*.	Distilled water	Flavonoids (rutin, myricetin and quercetin) and phenolic acids (gallic, caffeic and ferulic acid)	[10]
	Inhibition of *E. coli*, *S. aureus*, *P. aeruginosa*, *E. faecalis*, *S. enteritidis*, *C. albicans*, and bactericide against *P. aeruginosa*, *E. faecalis*, *S. enteritidis*.	Ethanol 70%	Phenolic compounds, flavonoids	[80]
	Inhibition of *S. aureus*, *S. epidermidis*, *E. coli*, *K. pneumoniae*, *S. abony* and *P. aeruginosa*.	Methanol	Phenolic compounds, flavonoids and anthocyanins	[78]
	Inhibition of methicillin-sensitive *Staphylococcus aureus* (MSSA) at a concentration of 2.5 mg/mL.	Ethanol 50%	Phenolic compounds, flavonoids and anthocyanins	[82]
	Blackthorn fruit peel is highlighted as a natural source of photosensitizers, and a potential solution is provided for the control of Listeria monocytogenes in food safety applications.	Ethanol	Polyphenolic compounds such as quercetin and rutin	[88]
Anti-inflammatory	Increased miR-146a and decreased expression levels of IRAK-1 and IL-6 with consequent downregulation of TLR-NF-κB mediated by inflammatory response, particularly by inhibiting the TLR4 signalling pathway and reducing cytokine production.	Ethanol 70% acidified with HCl	Phenolic compound	[11]
	Treatment with *P. spinosa* ethanol extract may prevent LPS stimulation by inhibiting TLR4 signalling and reducing the production of cytokines (IL-6 and TNFα) and cell adhesion molecules (ICAM-1 and VCAM-1).	Ethanol 70%	Phenolic compounds, flavonoids	[80]
	*P. spinosa* fruit extract plays an antioxidant role by decreasing ROS levels during inflammation when Nrf2 is activated and can prevent a pro-inflammatory response by inhibiting the TLR4/NF-kB mediated inflammatory cascade.	Ethanol	Phenolic compounds, flavonoids	[11]
Hypoglycaemic	Inhibitory capacity of extracts enriched in phenols of fresh and dried fruits of *P. spinosa* on glycolytic enzymes (α-glucosidase and α-amylase), related to Diabetes Mellitus (DM).	Methanol 75%	Polyphenolic components, such as flavonoids, anthocyanins, and phenolic acid derivatives and proanthocyanins	[12]
	Inhibition of α-glucosidase rather than α-amylase.	Methanol	Phenolic compounds, flavonoids, and anthocyanins	[78]
	Reduction of diabetes and obesity and postprandial glucose by inhibition of pancreatic α-amylase and intestinal α-glucosidase, and diminution of glucose transport in the small intestine and inhibition of glucose uptake in colorectal adenocarcinoma epithelial cells.	Not determined	Anthocyanins (cyanidin 3-O-glucoside and cyanidin 3-O-rutinoside)	[76]
Cellular senescence	The older cells showed a phenotype associated with pro-inflammatory and pro-oxidative senescence, that is, a pro-inflammatory condition characterized by high amounts of IRAK-1 and IL-6. After treatment with the extract, the modulation of miR-146a, IL-6 and IRAK-1 was comparable to what was observed in younger cells, acting as an anti-inflammatory agent.	Ethanol 70% acidified with HCl	Phenolic compound	[11]
Neuroprotective	Inhibition of AChE and tyrosinase.	Methanol	Not determined	[78]
	AChE and monoamine oxidase inhibition, diminution of peroxyl radical capture and oxidation, neurotrophic action, and maintenance of physiological functions of vital organs.	Not determined	Flavonoids (quercetin and rutin)	[76]
	Blackthorn skin extracts (1–10 micrograms per gram) inhibited beta-amyloid aggregation and protected against oxidative stress in SH-SY5Y cell lines.	Not determined	phenolic composition	[89]
UV photoprotection	Tyrosinase inhibitor.	Propylene glycol 45% (*v*/*v*)	Phenolic compounds, flavonoids, and anthocyanins	[78]
Antitumor	Inhibition of HCT116 cell growth and colony formation (35%) (2D and 3D models) compared to chemotherapy treatment with 5-fluorouracil (80%) used in clinical therapy.	Ethanolic extraction	Flavones, flavonols, phenolic acids and anthocyanins	[90]
	Cytotoxic activity on some cancer cell lines, inducing in vitro endogenous antioxidant mechanisms and modulating Nrf2 transcription factors, a regulator of cellular resistance to oxidative damage.	Not determined	Phenolic acids	[76]
Cardioprotective	Inhibition of pro-inflammatory enzymes, antiatherosclerotic and antithrombotic effect, modulation of lipid metabolism, improvement of capillary permeability and endothelial function with vasodilatory effects.	Not determined	Flavonoids	[76]
	Modulation of lipid metabolism, increasing antioxidant capacity of plasma, improving vascular functions, and decreasing platelet activity.	Not determined	Proanthocyanins	[76]
	Improve lipid decreasing mechanisms, antioxidant activity on ROS, antiglycation activity, and inhibition of different enzymes related to lipid metabolism, increase tissue tolerance to ischemia injury, reduce the risk of cardiovascular diseases, hypertension, have the capacity to scavenge ROS and decrease oxidative stress, enhancing inflammatory responses.	Not determined	Anthocyanins (cyanidin 3-O-rutinoside and cyanidin 3-O-glocoside)	[76]
In vivo studies				
Antioxidant	Reduction of hepatic and cerebral oxidative stress in rats.	Distilled water	Phenolic compounds (rutin, ferulic acid and trans-synaptic acid)	[10]
	Wild-type *Caenorhabditis elegans* worms treated with *P. spinosa* extract at the highest concentration (400 μg/mL) exhibited greater resistance to H_2_O_2_-induced oxidative stress compared to untreated worms.	Ethanol 70% acidified with HCl	Phenolic compound	[11]
Longevity study	Treatment of *C. elegans* with *P. spinosa* extract (400 μg/mL) significantly extended the half-life to 24.71 ± 0.48 days, and the survival rate increased by 22%.	Ethanol 70% acidified with HCl	Phenolic compound	[11]
MicroRNA modulation	The expression of miR-124 increased after treatment with *P. spinosa*, while miR-39 was reduced. Both results seem to suggest a positive potential in the lifespan of *C. elegans* by modulating the expression levels of miR-124 and miR-39.	Ethanol 70% acidified with HCl	Phenolic compound	[11]
Antitumor	Food supplement based on *P. spinosa* drupe extract combined with nutraceutical activator complex (NAC) slows the growth of colorectal cancer in mice.	Ethanolic extraction	Flavones, flavonols, phenolic acids and anthocyanins	[90]
Gastroprotective	Protection of the gastric mucosa was demonstrated through the modulation of inflammation and the PGE2 pathway by *P. spinosa*, with its effects shown to be dose-dependent and comparable to or more effective than the reference substance.	Ethanolic extraction	Phenolic compound	[91]

## Data Availability

No new data were created or analyzed in this study. Data sharing is not applicable to this article.
